# Effects of post-fermentation on the flavor compounds formation in red sour soup

**DOI:** 10.3389/fnut.2022.1007164

**Published:** 2022-10-28

**Authors:** Xiaojie Zhou, Wenhua Zhou, Xiaojie He, Yaxin Deng, Liangyi Li, Ming Li, Xuzhong Feng, Lin Zhang, Liangzhong Zhao

**Affiliations:** ^1^College of Food Science and Engineering, Central South University of Forestry and Technology, Changsha, China; ^2^College of Food and Chemical Engineering, Shaoyang University, Shaoyang, China; ^3^Hunan Key Laboratory of Processed Food for Special Medical Purpose, Changsha, China; ^4^Hunan Provincial Key Laboratory of Soybean Products Processing and Safety Control, Shaoyang, China; ^5^Shenzhen Shanggutang Food Development Co., Ltd., Shenzhen, China

**Keywords:** bacterial diversity, effect mechanism, post-fermentation, red sour soup (RSS), volatile compounds

## Abstract

Red Sour Soup (RSS) is a traditional fermented food in China. After two rounds of fermentation, sour soup has a mellow flavor. However, the microbial composition and flavor formation processes in post-fermentation in RSS are unclear. This study investigates the bacteria composition of RSS during the post-fermentation stage (0–180 days) using high-throughput sequencing. The results show that lactic acid bacteria (LAB) are dominant during the post-fermentation process, and their abundance gradually increases with fermentation time. Additionally, gas chromatography-mass spectrometry was used to detect volatile flavor compounds in the post-fermentation process. Seventy-seven volatile flavor compounds were identified, including 24 esters, 14 terpenes, 9 aromatic hydrocarbons, 9 alkanes, 6 heterocyclic compounds, 3 alcohols, 3 acids, 3 ketones, 2 phenols, 2 aldehydes, 1 amine, and 1 other. Esters and aromatic hydrocarbons are the main volatile compounds in RSS during the post-fermentation process. Orthogonal partial least squares screening and correlation analysis derived several significant correlations, including 48 pairs of positive correlations and 19 pairs of negative correlations. Among them, *Acetobacter* spp*., Clostridium* spp. and *Sporolactobacillus* spp. have 15, 14, 20 significant correlation pairs, respectively, and are considered the most important bacterial genera post-fermentation. Volatile substances become abundant with increasing fermentation time. LAB are excessive after more than 120 days but cause a drastic reduction in volatile ester levels. Thus, the post-fermentation time should be restricted to 120 days, which retains the highest concentrations of volatile esters in RSS. Overall, these findings provide a theoretical basis to determine an optimal post-fermentation time duration, and identify essential bacteria for manufacturing high-quality starter material to shorten the RSS post-fermentation processing time.

## Introduction

In China, fermentation has more than 3,000 years of history, and there are representative traditional fermented foods with unique characteristics for different regions or ethnic groups (1–3). Sour soup is a well-known traditional fermented food with a thousand years of history originating from a minority group in Guizhou of Kaili, China (4). It has been used as a condiment due to its unique flavor and high nutritional value. It can promote digestion, regulate intestinal flora, improve free radical scavenging and enhance immunity (5). Sour soup is categorized as Red Sour Soup (RSS) and white sour soup due to the differences in raw materials and fermentation process (6). RSS is mainly made of fresh red pepper and tomato, fermented for a few months by spontaneous fermentation without starter material (7). RSS’s widely used production technology includes two anaerobic fermentation stages: pre-fermentation and post-fermentation (8). Pre-fermentation is the separate fermentation process of tomato and pepper in a jar for 3 to 12 months. Post-fermentation is a mixture of pre-fermented tomatoes and peppers in proportion, with an appropriate number of auxiliary materials added, such as rice milk, and then put into a jar for fermentation for 1 to 6 months.

Like other fermented vegetables, the fermentation process of RSS involves complex microbial communities. Many studies demonstrated that the fermented food flavor is mainly attributed to the contribution of microbial communities in succession (2, 3, 9). When investigating the flavor components of commercial sour soup, it was found that predominantly volatile flavor compounds are esters and terpenoids (10–12). Esters are mainly derived from raw materials and the metabolites of microorganisms, while terpenoids mainly come from the raw material itself. Lactobacillus, Weissella and yeast were previously detected in RSS, with lactic acid bacteria (LAB) playing a leading role (13). LAB secretes large amounts of lactic acid, which not only participate in the reaction to produce abundant esters to enrich the volatile flavor but also generates extreme environmental pressure to inhibit the growth of miscellaneous bacteria and improve product quality. Additionally, some studies have found that fungi and bacteria played a role in the fermentation of sour soups. But, the presence of fungi seemed to incur the risk of some toxins that could produce an unpleasant flavor (9). However, the microbiota succession of RSS during post-fermentation and its relationship with the specific flavor of RSS remains unclear.

Various methods were undertaken to study bacterial diversity, including microbial isolation and cultivation, polymerase chain reaction-denaturing gradient gel electrophoresis (PCR-DGGE), and DNA cloning libraries (14–16). However, the conventional methods of microorganism investigation take time and have low sensitivity. In contrast, the high-throughput sequencing method has the advantages of short sequencing time and high sequencing flux, allowing it to determine the full diversity of microbial flora (17, 18). Headspace solid-phase microextraction coupled with gas chromatography-mass spectrometry (HS-SPME-GC-MS) with high sensitivity has been widely chosen to determine volatile compounds in samples (19, 20). Recently, these techniques have been used to detect microbial and flavor compounds in traditionally brewed foods (21, 22). Orthogonal partial least squares discriminant analysis (OPLS-DA) and Pearson correlation analysis have been widely applied to investigate the relationship between microorganisms and flavor substances in fermented foods (23, 24).

Currently, some studies explored the dynamic changes of microbial communities and flavor substances and the correlation between bacterial communities and flavor substances in RSS (8, 11, 12). However, most researchers focus on the kinds of bacteria and flavor compounds in RSS during the pre-fermentation process (7–9, 12). The correlation between bacterial successions and the production of volatile aromatic substances during the post-fermentation is unclear. This study performs HS-SPME-GC-MS to evaluate the correlation between bacterial communities and volatile aromatic substances in four different post-fermentation stages (0–180 days) of RSS. The results will enable the characterization of condiment fermentation mechanisms, while providing a theoretical framework for directional regulation of RSS.

## Materials and methods

### Sample collection and analysis

The RSS samples were provided by Kaili Lianghuan Biotechnology Co., Ltd (Guizhou, China). The RSS was manufactured according to Li et al. (8). The sample production process was: (1) mix pepper and distilled water with a ratio of 1:2 (wt/wt). Once smashed, add 30 g/kg of salt, followed by storage in fermenting jars for 30 days. (2) Fresh tomato was directly chopped and ground into a colloid sauce, 30 g/kg salt was added, and fermented in jars for 30 days. (3) Mix the finished fermentation products of (1) and (2) in a ratio of 1:3, then add 1% (wt/wt) rice milk, and transfer into the jar. The fermentation tank was then loaded, and the product was fermented for 180 days. Four groups of samples were collected at the appropriate fermentation time points (0 days, 60 days, 120 days, and 180 days) with six replicates per group. The test samples were from batches with different post-fermentation times and labeled sequentially as F-A (0 days), F-B (60 days), F-C (120 days) and F-D (180 days). Samples were prepared by mixing equal amounts of the mixture from five points, consisting of the four corners and the midpoint of the fermentation tank: upper (1 sample), middle (2 samples), left (1 sample), right (1 sample) and bottom (1 sample). The mixed samples were collected in sterilized sampling bottles. Twenty-four samples were collected in sterilized vials, transported on dry ice to the laboratory, and stored at –80°C for analysis.

### Illumina MiSeq sequencing

The total DNA from each 5 mL sample of sour soup was extracted using the E.Z.N.A.^®^ Soil DNA Kit (Omega Bio-Tek, Norcross, GA, United States) following instructions provided by the manufacturer. Agarose gel electrophoresis was performed to check DNA quality, and a NanoDrop 2000 UV-vis spectrophotometer (Thermo Scientific, Wilmington, USA) was used to measure the final DNA concentration and purity. Using a thermocycler PCR system (GeneAmp 9700, ABI, USA), primers 338F (5′-ACTCCTACGGGAGGCAGCAG-3′) and 806R (5′-GGACTACHVGGGTWTCTAAT-3′) were used to amplify the V3-V4 hypervariable regions of the bacterial 16S rDNA gene [Lin et al. (10)]. For the PCR reaction, the following program was used: 3 min of denaturation at 95°C, 27 cycles of 30 s each at 95°C, 30 s for annealing at 55°C, and 45 s for elongation at 72°C, followed by a final extension at 75°C for 5 min. This reaction was performed in triplicate in a 20 μL mixture containing 4 μL of 5 × FastPfu Buffer, 2 μL of 2.5 mmol/L dNTPs, 0.8 μL of each primer (5 μmol/L), 0.4 μL of FastPfu Polymerase and 10 ng of template DNA. The AxyPrep DNA Gel Extraction Kit (Axygen Biosciences, Union City, CA, USA) was used to extract PCR products from a 2% agarose gel and purify them further. The QuantiFluor™-ST system (Promega, USA) was used to quantify the DNA according to the manufacturer’s protocol. Purified amplicons were sequenced on an Illumina MiSeq platform (Illumina, San Diego, USA) using equimolar pooling and paired-end sequencing (2 × 300 bp). Quantitative library concentrations were measured using a Qubit v.3.0 Fluorometer (Invitrogen, Carlsbad, CA, USA). The library was quantified to 10 nmol/L. Illumina MiSeq (Illumina, San Diego, CA, USA) was used for the PE250/FE300 paired-end sequencing, and data were read using MiSeq Control Software (Illumina, San Diego, CA, USA).

### Volatile flavor component analysis

Volatile organic compounds (VOCs) of RSS samples were analyzed using HS-SPME-GC/MS according to previously reported methods (8) with some modifications. Samples (1 g) of RSS were immediately transferred to Agilent headspace vials (Agilent, Palo Alto, CA, USA) with a NaCl-saturated solution to inhibit enzyme reactions. Crimp-top caps with TFE-silicone septa (Agilent) were used to seal the vials. Each vial was incubated at 60°C for 10 min. Following this, a 65 μm carboxen-polydimethylsiloxane fiber (Supelco, Bellefonte, PA, USA) was inserted in the sample’s headspace for 20 min at 60°C. To extract the VOCs from the fiber coating, the injection port of a high-performance gas chromatography instrument (Model 7890B, Agilent) was heated to 250°C for 5 min in splitless mode after sampling. The identification and quantification of VOCs were undertaken using an Agilent Model 7890B GC and a 7000D mass spectrometer (Agilent), equipped with a 30 m × 0.25 mm × 1.0 μm DB-5MS (5% phenyl-polymethylsiloxane) capillary column. Helium (99.999% purity) was used as the carrier gas at a linear velocity of 1.0 mL/min. The injector temperature was maintained at 250°C and the detector at 280°C. The oven temperature was programmed from 40°C (maintained for 5 min) to 280°C (maintained for 5 min) with a rate of 6°C/min. Spectra were recorded at 70 eV using electron impact ionization mode (EI) and scanned in an m/z range of 30–350 amu at 1 s intervals. The temperatures of the quadrupole mass detector, ion source and transfer line were set at 150, 230, and 280°C, respectively. The volatile compounds were identified by comparing the mass spectra with the MetWare data system library.^1^

### Statistical analysis

Sequence clustering was performed using VSEARCH (v. 1.9.6) (the similarity level was set to 97%). Silva 132 was used as the 16S rRNA reference database. Species classification analysis of the operational taxonomic units (OTUs) was carried out using the RDP Classifier (Ribosomal Database Program) Bayesian algorithm. The alpha diversity indices were calculated based on the OTU analysis results. Principal component analysis (PCA) and Pearson’s correlation were performed with selected signals obtained through chromatogram processing in SPSS 22.0 (SPSS 22.0 for Windows, SPSS, Chicago, Illinois, USA).

## Results

### Overview of illumina MiSeq sequencing data for red sour soup samples

**FIGURE 1:**
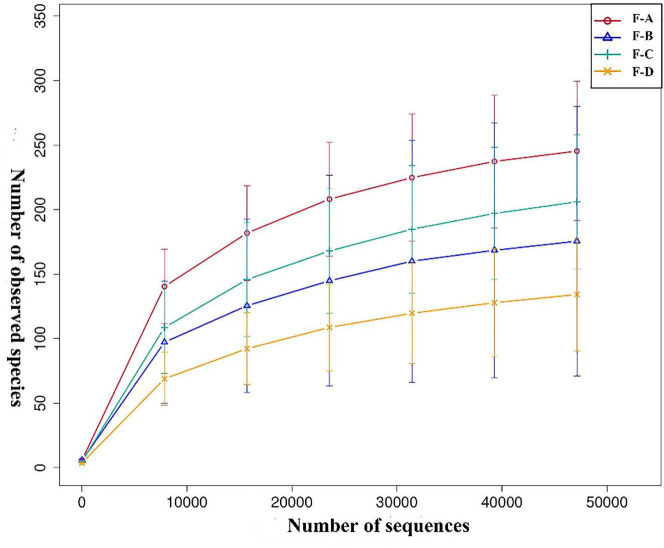
Rarefaction curve analysis of different sour soup samples at different post-fermentation stages. Each line of a different color represents data from each sample group. F-A, F-B, F-C and F-D refer to the sour soup samples fermented for 0 days, 60 days, 120 days, and 180 days, respectively. The abscissa denotes the number of sequences, and the ordinate denotes the number of observed OTUs.

Based on the 97% similarity of OTUs, Figure 1 shows the rarefaction curve of the bacterial community. Approximately 8,000 sequences provided sufficient coverage of all taxa in the four groups, and the number of observed OTUs stabilized beyond this point.

Under the 97% similarity threshold of OTUs, the alpha diversity indices of four sample groups were used to assess differences in the abundance and diversity of bacteria. The observed species, Shannon, Simpson, ACE, Chao1 and Good’s coverage indices are shown in Table 1. The results indicate that the Good’s coverage index of each group is equal to 1, indicating that the sequencing depth of all samples was sufficient to provide a reliable overall representation of the bacteria. The highest value of observed species is found in group F-C (120 days), followed by groups F-A (0 days) and F-D (180 days), and finally, F-B (60 days). Similar trends are found in the Chao1 and ACE indices. These results indicate that at 120 days of fermentation, the RSS has the highest bacterial richness. The Shannon and Simpson indices decrease gradually with increasing fermentation time, illustrating that the diversity of bacteria in RSS decreases with fermentation time.

**TABLE 1:** Sequencing data and alpha diversity analysis.

Groups	Observed species	Shannon	Simpson	Chao1	ACE	Good’s coverage
F-A	230.4 ± 25.29^a^	3.61 ± 0.41^a^	0.84 ± 0.03^a^	242.3 ± 27.23^a^	248.0 ± 26.54^a^	1 ± 0^a^
F-B	97.2 ± 14.03^b^	2.92 ± 0.58^ab^	0.76 ± 0.13^a^	103.2 ± 15.34^b^	106.3 ± 10.78^b^	1 ± 0^a^
F-C	231.7 ± 39.46^a^	2.85 ± 0.49^ab^	0.79 ± 0.03^a^	265.8 ± 32.21^a^	281.4 ± 30.26^a^	1 ± 0^a^
F-D	122.3 ± 16.27^b^	1.97 ± 0.09^b^	0.58 ± 0.01^b^	136.6 ± 13.46^b^	143.6 ± 18.03^b^	1 ± 0^a^

^1^F-A, F-B, F-C and F-D refer to the sour soup samples fermented for 0 days, 60 days, 120 days and 180 days, respectively. a, b, c, d, e, Statistical analysis was performed by one-way ANOVA (Tukey’s test, *P* < 0.05).

**FIGURE 2:**
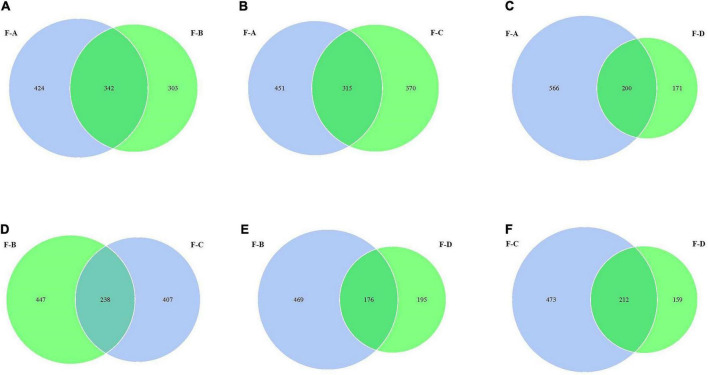
The Venn analysis of the bacterial composition in red sour soup based on OUT levels. Each circle represents a sample, the numbers of circles and overlapping parts represent the number of OTUs shared between samples, and the numbers without overlapping parts represent the number of unique OTUs of samples.

From Figure 2, the values of OTUs in RSS are significantly different at the four post-fermentation stages. The standard and unique OTUs drawn are represented using Venn diagrams, which more intuitively show the uniqueness and overlap of sample OTUs composition. The total OTUs in the F-A, F-B, F-C and F-D groups are 766, 645, 685 and 371, respectively. Figure 2 showed that their shared OTUs were 342, 315, 200, 238, 176, and 212 in the intersection of F-A and F-B, F-A and F-C, F-A and F-D, F-B and F-C, F-B and F-D, F-C and F-D, respectively. At the initial stage of post-fermentation (0–60 days), the number of intersectional OTUs was 342 (Figure 2A). Surprisingly, that gradually decreases to 315 and 200 when the fermentation time increases to 120 and 180 days (Figures 2B,C). According to Figures 2D–F, the uniqueness of OTUs mainly occurs between the 120 days and 180 days samples. At the other fermentation times, there are minimal changes in the uniqueness of OUTs.

### Microbiota community in red sour soup at different fermentation stages

**FIGURE 3:**
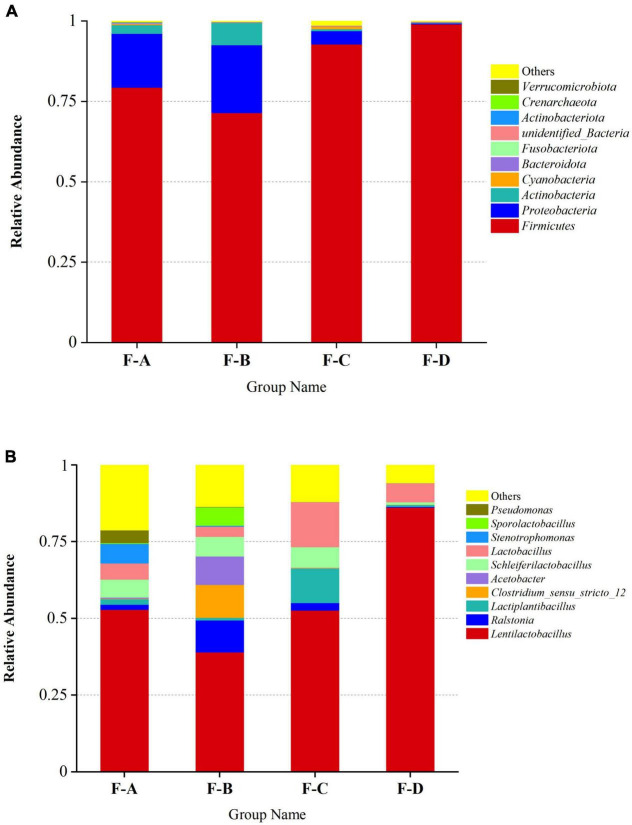
Relative abundance of bacteria in RSS samples at the phylum **(A)** and genus **(B)** levels at different post-fermentation stages. F-A, F-B, F-C and F-D refer to the sour soup samples fermented for 0 days, 60 days, 120 days, and 180 days, respectively. Each column represents a sample group containing a variety of bacteria.

The phylum and genus of the bacteria were distinguished based on the OTUs level to investigate the bacterial compositions in detail. More than 30 phyla and 364 genera are detected in RSS samples during post-fermentation, and the top 10 most abundant groups are shown in (Figure 3). Firmicutes (72.94–99.18%), Proteobacteria (0.16–23.16%) and Actinobacteriota (0.11–7.39%) are the dominant phyla in the fermentation process of sour soup, accounting for more than 90% (Figure 3A). Interestingly, Firmicutes decrease at 120 days and then increase with increasing fermentation time. Proteobacteria and Actinobacteriota exhibited a gradually decreasing trend during the post-fermentation process. Miscellaneous bacteria gradually decrease during fermentation, indicating that increasing the fermentation time can reduce the miscellaneous bacteria content.

At the genus level (Figure 3B), the main genera were detected at a high abundance (≥ 1% in at least one sample), including *Lactobacillus* spp*., Ralstonia* spp., *Clostridium* spp., *Acetobacter* spp., *Stenotrophomonas* spp., *Pseudomonas* spp., *Propionibacterium* spp., *Pediococcus* spp., *Halomonas* spp., and *Aliidiomarina* spp. Among them, LAB are the dominant bacteria (60–98%), consisting of *Lentilactobacillus* spp., *Lactiplantibacillus* spp., *Schleiferilactobacillus* spp., *Lactobacillus* spp., *Sporolactobacillus* spp., *Pediococcus* spp., *Limosilactobacillus* spp., *Loigolactobacillus* spp., *Levilactobacillus* spp., *Lacticaseibacillus* spp., *Companilactobacillus* spp., *Liquorilactobacillus* spp., *Ligilactobacillus* spp. and others, especially *Lentilactobacillus* spp. (39.02–86.23%). The levels of these bacteria decrease at 60 days (60.35%) and then increase with increasing post-fermentation time (120–180 days). The second-most abundant is *Ralstonia* spp., accounting for 0.17–10.41%. The content of *Ralstonia* spp. was high at the beginning of post-fermentation, especially at 60 days of fermentation (10.41%), and sharply decreased to 0.12–0.02% during the later stages. The levels of *Acetobacter* spp. and *Clostridium* spp. significantly increased after 60 days of fermentation time, accounting for 9.23% and 10.73%, respectively, but were lower at other fermentation points. The content of *Stenotrophomonas* spp. was high during the initial post-fermentation stage (0 days, 6.44%) but gradually decreased with fermentation and disappeared entirely at 180 days.

### Analysis of enrichment of metabolic pathways

**FIGURE 4:**
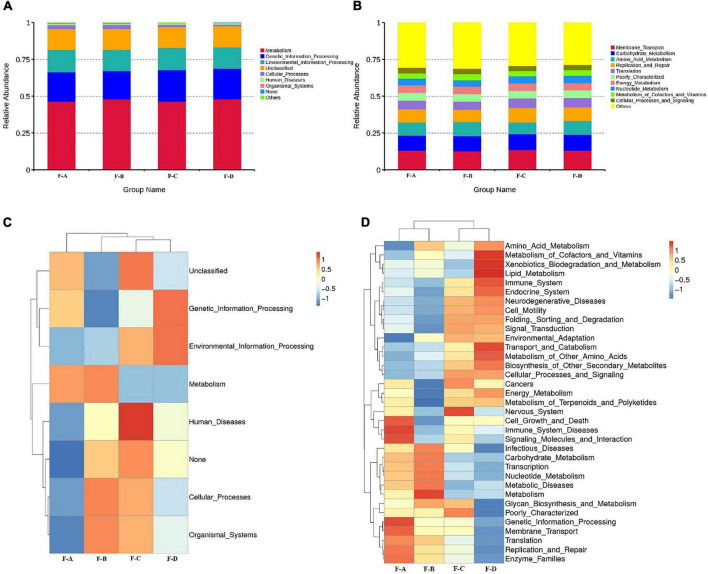
Abundance of Primary Function of bacteria at level 1 **(A)**, level 2 **(B)**, and cluster heatmap of Primary Function of bacteria at the level 1 **(C)**, and level 2 **(D)** by Picrust in RSS samples.

The Kyoto Encyclopedia of Genes and Genomes (KEGG) is an integrated database resource for associating genomic sequences with biological functions. Figure 4 shows the dynamic change of microbial function predicted for RSS during post-fermentation. Figure 4A shows the composition of level 1 KEGG pathways in bacteria from samples during different post-fermentation stages. The functionality of the bacteria in RSS is mainly for metabolic activity, including carbohydrates, amino acids, energy, nucleotide, cofactor, vitamin, terpenoid substances and poly ketone metabolism. The most important metabolic pathways are carbohydrates and amino acid metabolism. Second, glycan and other secondary metabolites that are involved in synthesis and metabolism (Supplementary Figure 1). These metabolic activities provide the unique flavor of RSS.

As shown in Figure 4D, the intensity of carbohydrate metabolism increases gradually with the post-fermentation time. It reaches the maximum at 180 days, which may be related to the abundance of LAB (25). Much evidence shows that the function of carbohydrate metabolism is mainly assigned to LAB in the vegetable fermentation process, and carbohydrate metabolism contributes to the survival of LAB in various environments (26–28). The metabolism of carbohydrates through glycolytic and citrate cycle pathways can produce acids and substrates for amino acid metabolism, as well as some by-products (29). The intermediate pyruvate is also a core compound in producing organic acids, ethanol and esters (30). Finally, the intensity of amino acid metabolism reaches a peak value at 60 days. The metabolic activity of terpenes decreased with fermentation time. Secondly, the abundance of gene annotations related to genetic information and environment process was high, and both reached the highest value at 120 days. Genetic annotations related to human disease have also been detected. However, with increasing fermentation times, the abundance of genetic annotations related to human disease gradually decreased and reached the lowest level at 180 days, consistent with decreasing miscellaneous bacteria content (Table 1).

### The composition of volatile compounds in red sour soup

Flavor substances are one of the critical indices to evaluate the quality of fermented foods. In the fermentation process, various substrates can be converted into distinctive metabolites through biocatalytic reactions of microorganisms, causing unique flavors in RSS. Thus, the RSS samples at four stages of the fermentation process between 0 and 180 days were investigated using HSPE-GC-MS. Table 2 shows a total of 77 kinds of volatile flavor compounds were detected in RSS samples, including 24 esters, 14 terpenes, 9 aromatic hydrocarbons, 9 alkanes, 6 heterocyclic compounds, 3 alcohols, 3 acids, 3 ketones, 2 phenols, 2 aldehydes, 1 amine, and 1 classified as other.

**TABLE 2:** Aroma compounds detected by GC-MS in different samples.

Classification	Compounds	RT	F-A	F-B	F-C	F-D
amines	m-Aminophenylacetylene	1.21E + 01	0.04 ± 0.000^a^	0.04 ± 0.005^a^	0.03 ± 0.000^a^	0.03 ± 0.005^a^
alcohols	3-methyl-1-butanol	3.09E + 00	6.26 ± 0.212^a^	5.83 ± 0.094^a^	2.29 ± 0.105^b^	4.99 ± 1.115^a^
	Benzyl alcohol	1.02E + 01	0.12 ± 0.005a	0.06 ± 0.046a	0.09 ± 0.006a	0.12 ± 0.005a
	6-methyl-5-hepten-2-ol	9.06E + 00	0.24 ± 0.000a	0.19 ± 0.015a	0.19 ± 0.027a	0.21 ± 0.065a
aromatic hydrocarbons	2-methoxyphenol	1.17E + 01	10.22 ± 0.404^c^	5.98 ± 0.151^d^	12.28 ± 0.173^b^	14.49 ± 0.424^a^
	Naphthalene	1.46E + 01	1.34 ± 0.207^a^	1.28 ± 0.076^a^	1.40 ± 0.058^a^	1.17 ± 0.175^a^
	4-ethyl-2-methoxyphenol	1.71E + 01	1.42 ± 0.062^c^	4.41 ± 0.064^a^	3.77 ± 0.112^b^	3.90 ± 0.185^b^
	Mesitylene	8.34E + 00	0.08 ± 0.026^a^	0.11 ± 0.005^a^	0.08 ± 0.006^a^	0.06 ± 0.005^a^
	1-methyl-3-(1-methylethyl)-benzene	9.96E + 00	1.33 ± 0.139^b^	1.16 ± 0.067^b^	1.74 ± 0.085^b^	2.66 ± 0.504^a^
	1-ethenyl-3,5-dimethylbenzene	1.19E + 01	0.62 ± 0.065^b^	0.56 ± 0.065^b^	0.69 ± 0.027^b^	0.97 ± 0.115^a^
	pentamethylbenzene	1.53E + 01	0.45 ± 0.225^a^	0.19 ± 0.087^ab^	0.29 ± 0.086^ab^	0.09 ± 0.005^b^
	1,2,3,4-tetrahydro-1,4,6-trimethylnaphthalene	1.54E + 01	0.29 ± 0.046^b^	0.41 ± 0.087^b^	1.17 ± 0.025^a^	0.33 ± 0.017^b^
	2-ethylbenzene-1,4-diol	1.47E + 01	3.68 ± 0.158^a^	4.11 ± 0.446^a^	1.66 ± 0.027^b^	2.11 ± 0.055^b^
phenols	Eugenol	1.92E + 01	5.58 ± 0.153^c^	8.13 ± 0.354^a^	6.68 ± 0.261^b^	4.12 ± 0.139^d^
	5-pentyl-1,3-benzenediol	2.34E + 01	0.26 ± 0.005^b^	0.31 ± 0.036^a^	0.12 ± 0.006^c^	0.08 ± 0.005^c^
other	4-chlorobutanoic anhydride	1.91E + 01	0.12 ± 0.005^b^	0.18 ± 0.026^a^	0.11 ± 0.015^b^	0.15 ± 0.006^ab^
aldehydes	Benzaldehyde	8.11E + 00	0.76 ± 0.026^c^	0.56 ± 0.025^d^	0.88 ± 0.026^b^	1.57 ± 0.085^a^
	2,4-dimethylbenzaldehyde	1.55E + 01	7.00 ± 0.667^a^	2.23 ± 0.101^c^	4.99 ± 0.172^b^	5.42 ± 0.655^b^
acids	Non-anoic acid	1.72E + 01	2.39 ± 0.304^a^	2.75 ± 0.456^a^	2.02 ± 0.147^a^	3.00 ± 0.565^a^
	Octanoic acid	1.45E + 01	0.68 ± 0.031^c^	1.25 ± 0.048^b^	1.29 ± 0.046^b^	2.19 ± 0.037^a^
	Butanoic acid	3.98E + 00	0.06 ± 0.046^a^	0.16 ± 0.041^a^	0.12 ± 0.005^a^	0.02 ± 0.037^a^
terpenes	3-Carene	1.06E + 01	0.28 ± 0.025^c^	0.22 ± 0.017^d^	0.40 ± 0.028^b^	0.60 ± 0.035^a^
	gamma-Terpinene	1.10E + 01	0.07 ± 0.005^c^	0.07 ± 0.005^c^	0.10 ± 0.009^b^	0.18 ± 0.006^a^
	(E)-1-(2,6,6-trimethyl-1,3-cyclohexadien-1-yl)-2-buten-1-on	1.99E + 01	1.68 ± 0.005^c^	2.16 ± 0.167^b^	1.83 ± 0.066^c^	2.51 ± 0.109^a^
	D-limonene	1.01E + 01	0.69 ± 0.058^b^	0.65 ± 0.019^b^	0.80 ± 0.005^b^	1.18 ± 0.155^a^
	trans-beta-ionone	1.81E + 01	0.33 ± 0.016^b^	0.05 ± 0.005^b^	0.03 ± 0.005^b^	6.75 ± 0.587^a^
	(S)-(+)-alpha-phellandrene	9.42E + 00	0.28 ± 0.006^b^	0.23 ± 0.017^c^	0.25 ± 0.006^c^	0.36 ± 0.015^a^
	(+)-alpha-pinene	1.03E + 01	0.07 ± 0.000^c^	0.06 ± 0.016^c^	0.27 ± 0.025^b^	0.36 ± 0.025^a^
	(1S)-6,6-dimethyl-2-methylene-bicyclo[3.1.1]heptane	8.95E + 00	0.46 ± 0.026^c^	0.40 ± 0.035^c^	1.05 ± 0.068^b^	1.36 ± 0.202^a^
	2,6,10,10-tetramethyl-1-oxaspiro[4.5]dec-6-ene	1.78E + 01	1.62 ± 0.101^b^	2.54 ± 0.104^a^	2.42 ± 0.163^a^	0.09 ± 0.005^c^
	4-isopropyl-6-methyl-1-methylene-1,2,3,4-tetrahydronaphthalene	2.67E + 01	0.03 ± 0.000^ab^	0.03 ± 0.006^ab^	0.02 ± 0.000^b^	0.04 ± 0.005^a^
	2,6-dimethyl-2,4,6-octatriene	1.18E + 01	0.22 ± 0.005^c^	0.20 ± 0.005^c^	0.27 ± 0.007^b^	0.38 ± 0.036^a^
	alpha-dehydro-ar-himachalene	2.36E + 01	0.01 ± 0.000^c^	0.01 ± 0.000^b^	0.01 ± 0.000^a^	0.01 ± 0.000^c^
	Ionone	2.30E + 01	0.91 ± 0.005^b^	3.07 ± 0.217^a^	0.75 ± 0.036^b^	0.07 ± 0.001^c^
	1, 1, 5-trimethyl-1, 2-dihydronaphthalene	2.06E + 01	0.35 ± 0.026^b^	0.46 ± 0.035^b^	1.21 ± 0.058^a^	1.15 ± 0.136^a^
ketones	Isophorone	1.10E + 01	0.13 ± 0.002^b^	0.12 ± 0.006^b^	0.13 ± 0.015^b^	0.17 ± 0.016^a^
	Tropinone	1.06E + 01	0.08 ± 0.005^a^	0.08 ± 0.005^ab^	0.07 ± 0.001^b^	0.07 ± 0.001^ab^
	1-(4-hydroxy-3-thienyl)-ethanone	8.38E + 00	0.69 ± 0.105^a^	0.71 ± 0.076^a^	0.62 ± 0.037^a^	0.63 ± 0.105^a^
alkanes	Tetradecane	2.06E + 01	0.07 ± 0.004^a^	0.07 ± 0.005^a^	0.06 ± 0.005^a^	0.06 ± 0.006^a^
	Pentadecane	2.31E + 01	4.41 ± 0.121^a^	4.89 ± 0.405^a^	3.38 ± 0.073^b^	4.45 ± 0.527^a^
	Hexadecane	2.54E + 01	0.09 ± 0.005^b^	0.11 ± 0.005^a^	0.07 ± 0.001^b^	0.08 ± 0.006^b^
	Heptadecane	2.76E + 01	0.29 ± 0.026^ab^	0.32 ± 0.007^a^	0.23 ± 0.006^c^	0.25 ± 0.027^bc^
	Octadecane	2.98E + 01	0.08 ± 0.005^a^	0.08 ± 0.006^a^	0.08 ± 0.004^a^	0.08 ± 0.005^a^
	Heneicosane	2.75E + 01	0.03 ± 0.005^a^	0.04 ± 0.005^a^	0.03 ± 0.005^a^	0.03 ± 0.001^a^
	2,6,10-trimethyl-tetradecane	2.27E + 01	0.05 ± 0.003^b^	0.08 ± 0.005^a^	0.05 ± 0.001^b^	0.06 ± 0.005^b^
	2-methyl-pentadecane	2.46E + 01	0.01 ± 0.001^b^	0.02 ± 0.001^a^	0.01 ± 0.002^b^	0.02 ± 0.001^b^
	2,6,11,15-tetramethyl-hexadecane	2.38E + 01	0.14 ± 0.026^a^	0.14 ± 0.007^a^	0.12 ± 0.005^a^	0.12 ± 0.006^a^
heterocyclic compounds	2-isobutylthiazole	1.02E + 01	2.01 ± 0.036^a^	2.84 ± 0.315^b^	3.39 ± 0.058^a^	2.83 ± 0.206^b^
	7,9-di-tert-butyl-1-oxaspiro(4,5)deca-6,9-diene-2,8-dione	3.18E + 01	0.18 ± 0.055^a^	0.19 ± 0.055^a^	0.21 ± 0.027^a^	0.13 ± 0.055^a^
	4H-pyran-4-one	8.91E + 00	1.45 ± 0.076^a^	1.23 ± 0.048^a^	1.18 ± 0.079^a^	1.28 ± 0.215^a^
	1-(2-furanyl)-1-propanone	1.03E + 01	0.36 ± 0.025^a^	0.33 ± 0.027^ab^	0.28 ± 0.026^b^	0.33 ± 0.029^ab^
	4-isobutylpyrimidine	9.36E + 00	0.14 ± 0.015^a^	0.12 ± 0.005^a^	0.11 ± 0.003^a^	0.14 ± 0.026^a^
	2,4a-epidioxy-5,6,7,8-tetrahydro-2,5,5,8a-tetramethyl-2H-1-benzopyran	1.67E + 01	0.07 ± 0.002^b^	0.02 ± 0.005^b^	0.00 ± 0.003^b^	1.22 ± 0.101^a^
esters	Isobutyl acetate	3.39E + 00	0.23 ± 0.015^a^	0.17 ± 0.006^b^	0.24 ± 0.017^a^	0.03 ± 0.004^c^
	Propanoic acid, propyl ester (Propyl propionate)	4.32E + 00	6.00 ± 0.143^a^	3.64 ± 0.139^c^	4.65 ± 0.272^b^	1.16 ± 0.136^d^
	1-Butanol, 3- methyl-, acetate (Isoamyl acetate)	5.85E + 00	1.03 ± 0.062^c^	1.82 ± 0.127^b^	1.79 ± 0.108^b^	2.77 ± 0.404^a^
	Hexanoic acid, ethyl ester (Ethyl hexanoate)	9.22E + 00	0.03 ± 0.002^a^	0.04 ± 0.001^a^	0.04 ± 0.003^a^	0.03 ± 0.001^a^
	Propanoic acid, 2- hydroxy-, ethyl ester (Ethyl lactate)	6.66E + 00	0.58 ± 0.023^a^	0.19 ± 0.064^b^	0.33 ± 0.035^ab^	0.45 ± 0.148^ab^
	Butanedioic acid, diethyl ester (Diethyl succinate)	1.45E + 01	0.23 ± 0.006^d^	1.09 ± 0.085^b^	0.81 ± 0.016^c^	1.44 ± 0.202^a^
	Benzoic acid, ethyl ester (Ethyl benzoate)	1.42E + 01	0.12 ± 0.005^b^	0.15 ± 0.037^b^	0.14 ± 0.005^b^	0.83 ± 0.007^a^
	Methyl salicylate	1.48E + 01	16.26 ± 0.282^b^	10.38 ± 0.115^c^	17.62 ± 0.184^a^	9.32 ± 0.767^d^
	Linalyl acetate	1.22E + 01	0.45 ± 0.036^b^	0.41 ± 0.095^b^	0.53 ± 0.037^b^	1.06 ± 0.118^a^
	alpha-Terpinyl acetate	1.50E + 01	0.37 ± 0.045^a^	0.30 ± 0.057^a^	0.25 ± 0.076^a^	0.32 ± 0.027^a^
	Benzoic acid, 2- hydroxy-, ethyl ester (Ethyl salicylate)	1.70E + 01	0.81 ± 0.045^c^	0.28 ± 0.016^d^	1.07 ± 0.035^b^	1.44 ± 0.038^a^
	Dodecanoic acid, ethyl ester (Ethyl laurinate)	2.52E + 01	0.05 ± 0.001^b^	0.39 ± 0.045^a^	0.03 ± 0.001^b^	0.03 ± 0.005^b^
	Methyl tetradecanoate	2.81E + 01	0.01 ± 0.002^b^	0.02 ± 0.001^a^	0.01 ± 0.004^b^	0.01 ± 0.003^b^
	Tetradecanoic acid, ethyl ester (Ethyl myristate)	2.96E + 01	0.09 ± 0.015^b^	0.50 ± 0.046^a^	0.04 ± 0.003^b^	0.07 ± 0.003^b^
	Hexadecanoic acid, ethyl ester (Ethyl cetylate)	3.36E + 01	4.30 ± 0.765^b^	10.49 ± 0.758^a^	2.01 ± 0.186^c^	1.49 ± 0.285^c^
	Isopropyl palmitate	3.54E + 01	0.36 ± 0.057^b^	0.50 ± 0.116^a^	0.16 ± 0.015^c^	0.04 ± 0.006^c^
	Hexadecanoic acid, methyl ester (Methyl palmitate)	3.23E + 01	0.34 ± 0.045^b^	0.82 ± 0.066^a^	0.34 ± 0.009^b^	0.46 ± 0.075^b^
	Isopropyl myristate	3.16E + 01	0.02 ± 0.004^b^	0.06 ± 0.003^a^	0.01 ± 0.004^c^	0.01 ± 0.001^d^
	Ethyl 9-hexadecenoate	3.32E + 01	0.01 ± 0.002^b^	0.05 ± 0.005^a^	0.01 ± 0.003^c^	0.01 ± 0.002^c^
	n-propyl acetate	2.78E + 00	6.24 ± 0.48^b^	6.11 ± 0.456^b^	7.73 ± 0.535^a^	3.52 ± 0.386^c^
	Phthalic acid, butyl hex-3-yl ester	3.28E + 01	1.73 ± 0.092^a^	0.62 ± 0.165^b^	0.39 ± 0.025^c^	0.43 ± 0.027^c^
	(Z, Z)-9.12-octadecadienoic acid, methyl ester (methyl linoleate)	3.83E + 01	0.02 ± 0.001^b^	0.03 ± 0.002^a^	0.00 ± 0.000^c^	0.00 ± 0.000^c^
	Linoleic acid ethyl ester (Ethyl Linoleate)	3.66E + 01	0.04 ± 0.006^b^	0.10 ± 0.005^a^	0.01 ± 0.002^c^	0.01 ± 0.001^c^
	Ethyl 13-methyl-tetradecanoate	3.09E + 01	0.48 ± 0.021^a^	0.47 ± 0.055^a^	0.44 ± 0.037^a^	0.43 ± 0.026^a^

^2^ RT refers to the retention time. F-A, F-B, F-C and F-D refer to the sour soup samples fermented for 0 days, 60 days, 120 days and 180 days, respectively. Statistical analysis was performed using one-way ANOVA (Tukey’s test, *P* < 0.05).

**FIGURE 5:**
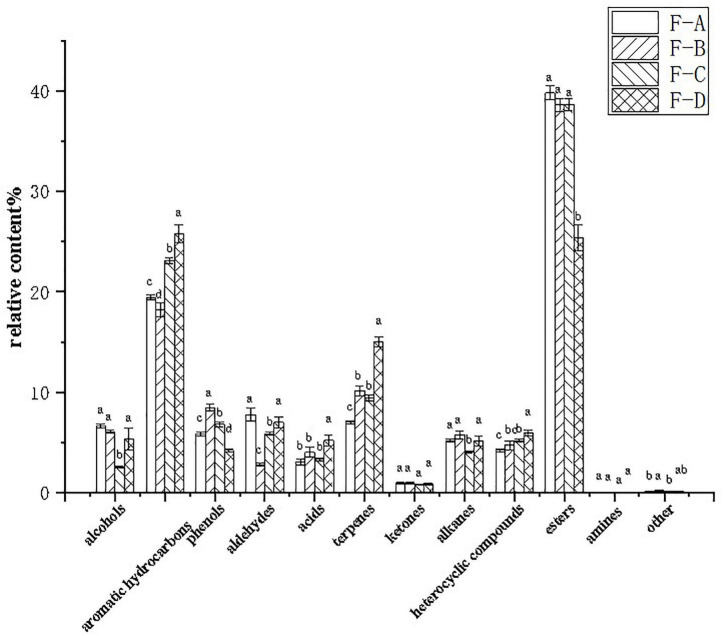
Relative percentages of volatile compounds in RSS samples at different post-fermentation stages. F-A, F-B, F-C, and F-D refer to the sour soup samples fermented for 0 days, 60 days, 120 days and 180 days, respectively. Each column represents a variety of substances in each sample. Statistical analysis was performed by one-way ANOVA (Tukey’s test, *P* < 0.05).

Figure 5 shows that esters are the dominant volatile substances in RSS during the post-fermentation process. The percentage of esters between 0 and 120 days of post-fermentation was insignificant, but it dramatically decreased to 25.36% from approximately 39.80% at 180 days. Furthermore, aromatic hydrocarbons (18.19–25.79%), terpenoids (6.99–15.01%), and heterocyclic compounds (4.21–5.92%) increased gradually during post-fermentation. Alcohols (2.57–6.08%) and alkanes (4.03–5.74%) suddenly decreased at 120 days of fermentation, while aldehydes (2.79–7.76%) suddenly decreased at 60 days and recovered to higher levels at 180 days (6.99%). Phenolic substances (4.20–8.44%) peaked at 60 days (8.44%), initially increased with fermentation time (0–60 days) and then decreased significantly (60–180 days).

Esters are abundant in the RSS fermentation process and usually have floral and fruity smells (12, 31). The high esters content includes propanoic acid propyl ester (propyl propionate), 1-butanol 3-methyl-acetate (isoamyl acetate), methyl salicylate, hexadecanoic acid ethyl ester (ethyl cetylate), n-propyl acetate, methyl salicylate and butyl hex-3-yl ester phthalic acid. Propyl propionate and butyl hex-3-yl ester phthalic acid levels decrease over time, while isoamyl acetate and benzoic acid, 2- hydroxy-, ethyl ester (Ethyl salicylate) increase with post-fermentation time. Ethyl cetylate and n-propyl acetate initially showed an increasing trend but then decreased, peaking at 60 and 120 days of fermentation, respectively. Finally, methyl salicylate had a decreasing trend before 60 days and then increased up to the 120-day point.

The aromatic hydrocarbon compounds also account for many of the total volatile compounds. Among them, 2-methoxyphenol, naphthalene, 4-ethyl-2-methoxyphenol, 1-methyl-3-(1-methylethyl)-benzene and 2-ethylbenzene-1,4-diol occurred at high percentages. 2-methoxyphenol exhibited a decreasing trend before 60 days and then increased, while it was inverse to 4-ethyl-2-methoxyphenol. The concentration of 1-methyl-3-(1-methylethyl)-benzene increased during the post-fermentation time, while that of 2-ethylbenzene-1,4-diol was the opposite. Terpenes are often derived from plants, and the most abundant terpenes included (E)-1-(2,6,6-trimethyl-1,3-cyclohexadien-1-yl)-2-buten-1-one, D-limonene, trans-beta-ionone, (1S)-6, 6-dimethyl-2-methylene-bicyclo[3.1.1]heptane, 2,6,10,10-tetramethyl-1-oxaspiro[4.5]dec-6-ene, and ionone. (E)-1-(2,6,6- trimethyl-1,3-cyclohexadien-1-yl)-2-buten-1-one fluctuated with post-fermentation and reached a peak at 180 days. Meanwhile, D-limonene, trans-beta-ionone and (1S)-6,6-dimethyl-2-methylene-bicyclo[3.1.1]heptane increased with fermentation time. 2,6,10,10-tetramethyl-1-oxaspiro[4.5]dec-6-ene first increased and then decreased. Other substances present at high contents included 2-isobutylthiazol, 4H-pyran-4-one, 3-methyl-1-butanol and pentadecane.

### Principal component analysis analysis of volatile compounds

**FIGURE 6:**
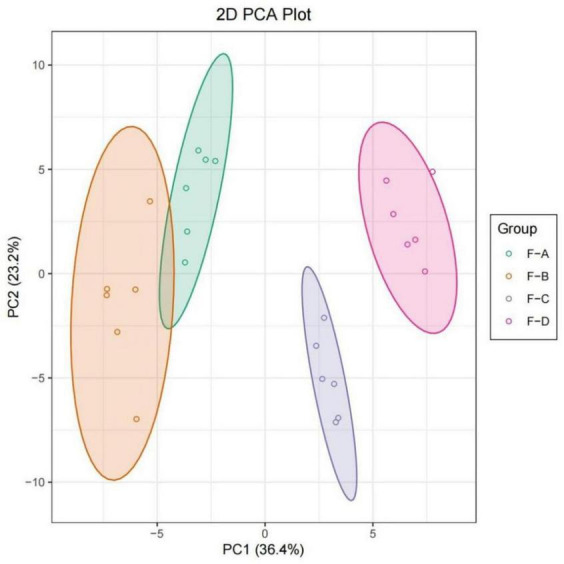
PCA plot based on HS-SPME-GC/MS results for RSS from different post-fermentation stages. F-A, F-B, F-C and F-D refer to the sour soup samples fermented for 0 days, 60 days, 120 days, and 180 days, respectively.

Principal component analysis (PCA) analysis was undertaken to compare the differences of volatile substances in RSS with different fermentation days. As shown in Figure 6, different samples were distinguished, and parallel samples were pooled. According to the first principal component, F-A and F-B were close together, indicating they were similar and clustered into one category. Taking the second principal component into account, all samples were relatively close. Therefore, the differences between these samples can primarily be explained using the first principal component. As can be seen from the overall distance, the changes in volatile substances were relatively small in the early stage of post-fermentation (0–60 days) and became more extensive in the later stage of post-fermentation (120–180 days).

### Correlation between microbiota and volatile components of red sour soup

**FIGURE 7:**
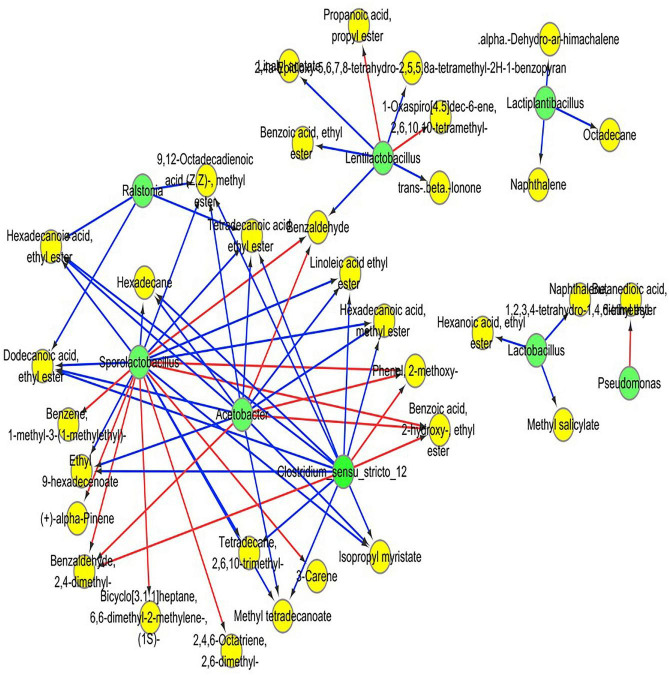
Heatmap of correlation analysis between dominant bacteria and some highly differential volatile components, based on Pearson’s correlation coefficients. The red line shows a positive correlation, and the blue line shows a negative correlation. Green circles indicate microorganisms, and yellow circles indicate volatile substances.

Pearson correlation analysis was employed to evaluate the relationship between the dominant bacteria and the predominant volatile components in RSS. OPLS was applied to screen the critical volatile components with variable importance in projection (VIP) values greater than 1. Pearson correlation analysis was undertaken for RSS’s top 10 most abundant bacteria and the main volatile compounds. Cytoscope software was used to portray the results through a visualization network. As shown in Figure 7, 67 significant correlations are identified. *Acetobacter* spp., *Clostridium* spp. and *Sporolactobacillus* spp. have over ten significant correlations, especially *Sporolactobacillus* spp., and all three bacteria are primarily correlated with esters. *Acetobacter* mainly correlated with benzoic acid, 2- hydroxy-, ethyl ester (ethyl salicylate), (Z, Z)-9.12-octadecadienoic acid, methyl ester (methyl linoleate), hexadecanoic acid, methyl ester (methyl palmitate), methyl tetradecanoate, dodecanoic acid, ethyl ester (ethyl laurinate), hexadecanoic acid, ethyl ester (ethyl cetylate), tetradecanoic acid, ethyl ester (ethyl myristate), isopropyl myristate, linoleic acid ethyl ester (ethyl linoleate) and ethyl 9-hexadecenoate. *Clostridium* spp. mainly correlate with benzoic acid, 2- hydroxy-, ethyl ester (ethyl salicylate), (Z, Z)-9.12-octadecadienoic acid, methyl ester (methyl linoleate), hexadecanoic acid, methyl ester (methyl palmitate), methyl tetradecanoate, hexadecanoic acid, ethyl ester (ethyl cetylate), dodecanoic acid, ethyl ester (ethyl laurinate), tetradecanoic acid, ethyl ester (ethyl myristate), isopropyl myristate, linoleic acid ethyl ester (ethyl linoleate), and ethyl 9-hexadecenoate. *Sporolactobacillus* spp. mainly correlate with benzoic acid, 2- hydroxy-, ethyl ester (ethyl salicylate), (Z, Z)-9.12-octadecadienoic acid, methyl ester (methyl linoleate), hexadecanoic acid, methyl ester (ethyl cetylate), methyl tetradecanoate, hexadecanoic acid, ethyl ester (ethyl cetylate), dodecanoic acid, ethyl ester (ethyl laurinate), tetradecanoic acid, ethyl ester (ethyl myristate), isopropyl myristate, linoleic acid ethyl ester (ethyl linoleate) and ethyl 9-hexadecenoate. *Acetobacter*, *Clostridium* spp. and *Sporolactobacillus* spp. also correlate with 2-methoxyphenol, 2,4-dimethyl-benzaldehyde, and hexadecane.

## Discussion

Red sour soup (RSS) is a traditional Chinese fermented food with a history of several thousand years (32). Its fermentation process relies mainly on LAB, acetic acid bacteria and *Clostridium* spp., which produce a refreshingly sour taste. As reported in previous studies, naturally fermented sour soup has more flavor after a longer fermentation time (more than 87 days) (33). However, for practical purposes, commercial production generally shortens the production period to 1–2 months by adding dominant strains (34, 35). This study aims to investigate the effect of fermentation time on the quality of RSS, the microbial composition and volatile flavor substances in RSS during the post-fermentation process.

Lactic acid bacteria (LAB) is commonly chosen as a starter material in various vegetable fermentation processes, such as Sichuan paocai, Korean kimchi, German sauerkraut and North-eastern Chinese pickles (36–39). LAB could convert fermentable sugars into lactic acid and other organic acids (40), which could help to produce flavor compounds in foods through fermentation (41). The gradual accumulation of acids will inhibit the growth of other microorganisms so that LAB gradually become the dominant bacteria (42, 43). Meanwhile, the diversity of LAB may also contribute to the production of volatile flavored compounds (13). As shown in Figure 3, LAB is the main bacteria in RSS in the post-fermentation process, consistent with sour soup’s widely reported microbial composition (4, 12, 13, 31, 32, 44). Notably, the central populations of LAB are *Sporolactobacillus* spp., *Lentilactobacillus* spp., *Lactiplantibacillus* spp., and *Lactobacillus* spp. *Sporolactobacillus* spp. predominantly correlate with 2- hydroxy-, ethyl ester, methyl ester, methyl ester, ethyl ester, ethyl ester, ethyl ester, linoleic acid ethyl ester, benzoic acid, hexadecanoic acid, and other volatile flavor compounds. *Lentilactobacillus* spp. is mainly related to benzoic acid ethyl ester, linalyl acetate, and propanoic acid propyl ester. *Lactiplantibacillus* spp. mainly correlate with α-deshydro-ar-himachalene, naphthalene, and octadecane, while *Lactobacillus* spp. mainly correlate with hexanoic acid ethyl ester and methyl salicylate. However, as Lin et al. reported, excessive LAB may inhibit the production of flavor substances, especially esters (12). The current study report that the lactic acid bacteria reached the highest content at 180 days, while the ester content decreased. A hypothesis was suggested that the underlying reason could relate to excess LAB inhibiting the growth of yeast and microorganisms related to ester production. The growth of yeast can produce many alcohols as precursors of esters (45). LAB inhibits yeast growth by producing bacteriostatic substances or robbing trace nutrients for fungal growth (46, 47).

*Acetobacter* spp. can produce acetic acid and are ubiquitous in the fermentation of various kinds of vinegar (48). Acetic acid production significantly contributes to the generation of esters from alcohols and can make the fermented product more aromatic (49). In addition, there is evidence that acetic acid bacteria can increase the total fatty acid content of the fermentation by breaking down amino acids (50). Pearson correlation analysis results indicate that in the post-fermentation process of RSS, *Acetobacter* spp. is positively correlated with a variety of esters, including (Z,Z)-9.12-octadecadienoic acid-methyl ester, 2-hydroxy-benzoic acid ethyl ester, dodecanoic acid ethyl ester, ethyl 9-hexadecenoate, hexadecanoic acid ethyl ester, hexadecanoic acid methyl ester, isopropyl myristate, linoleic acid ethyl ester, methyl tetradecanoate, and tetradecanoic acid ethyl ester.

*Clostridium* spp. are usually found in wine fermentation (51, 52). Acetyl coenzyme A can be synthesized via the reverse β-oxidation pathway by *Clostridium* spp. and then converted to butyric acid. Octanoic acid can be synthesized through a similar metabolic pathway by *Clostridium* spp. (53). Low concentrations of butyric acid are essential flavor compounds in many foods. However, the high concentration of butyric acid would create an unpleasant smell. Therefore, butyric acid is best kept at a low concentration during fermentation (54). The current study also detected butyric and caprylic acids as volatile flavor compounds.

Small amounts of *Ralstonia* spp. were also detected. *Ralstonia* spp. are usually found in soil and presumably were introduced by raw materials during production (55). In some cases, *Ralstonia* spp. are pathogenic and causes bacterial infections, and levels were inhibited later during fermentation (180 days) (56). Meanwhile, esters positively related with *Ralstonia* spp., such as (Z,Z)-9.12-octadecadienoic acid, methyl ester, dodecanoic acid ethyl ester, hexadecanoic acid ethyl ester, and tetradecanoic acid ethyl ester also decreased significantly at 180 days. However, its role in fermentation is unclear.

Hydrocarbons, including alkanes and aromatic hydrocarbons, were also detected during fermentation. Aromatic hydrocarbons have a unique aroma, but aromatic hydrocarbon compounds have a higher flavor threshold and may not significantly affect flavor. Nevertheless, at a specific concentration, they give fermented products a fuller flavor (57). Terpenoids are mainly produced by plants and have floral and woody scents. During fermentation, terpenoids are released due to the transformation of microorganisms, increasing terpenoid levels. LAB has been shown to release potential volatile terpenes through enzymatic hydrolysis (58–68). There may be a positive correlation between terpenoids’ content and consumers’ affection (60). High concentrations of aldehydes are often thought to be the unpleasant taste of vegetable fermentation, and lactic fermentation usually reduces the levels of related aldehydes (61). The current study found that aldehydes are negatively correlated with bacterial levels. Benzaldehyde and 2-4 dimethylbenzaldehyde are detected in our study, and give the fermentation desirable sensory properties, such as almond, bitter almond, cherry and sweet tastes (62, 63).

After a long post-fermentation process, the contents of volatile compounds, especially esters, acids, terpenes and aromatic compounds, were more abundant, indicating that a longer fermentation time was beneficial to expanding the volatile flavor. However, the data illustrate that when fermentation time is prolonged and between 120 and 180 days, the contents of the esters decrease. This may be due to the excessive LAB, which could inhibit the production of flavor substances (11). Due to the low flavor threshold of esters, even small changes in the concentrations of esters can directly impact the sour soup’s sensory quality (49). Thus, these results indicate that the optimal post-fermentation time is around 120 days in the RSS preparation process.

## Conclusion

This study investigated the relationship between microbial composition and volatile flavor substances in RSS during the post-fermentation process. *Acetobacter* spp*., Clostridium* spp. and *Sporolactobacillus* spp. influenced volatile flavor compound levels significantly. In addition, with the passage of fermentation time (0–120 days), the amounts of LAB and volatile flavor substances accumulate. Unfortunately, although LAB are overabundant in the lengthy post-fermentation time (180 days), esters production in RSS are inhibited. Thus, the fermentation time of RSS should be controlled at around 120 days. Notably, *Acetobacter* spp*., Clostridium* spp. and *Sporolactobacillus* spp. can be considered starter material for RSS fermentation and warrants further research.

## Data availability statement

The data presented in this study are deposited in the NCBI repository, accession number: PRJNA857173.

## Author contributions

XZ: writing—original draft and conceptualization. WZ: conceptualization. LZ and LZZ: writing—review and editing. ML and XF: visualization. XH, YD, and LL: software and formal analysis. All authors contributed to the article and approved the submitted version.

## Footnotes

1
http://www.metware.cn/
