# Supplementary figures and images for: Effects of post-fermentation on the flavor compounds formation in red sour soup
Source: Front Nutr. 2022 Oct 28;9:1007164. doi: 10.3389/fnut.2022.1007164 (PMC9651139; doi:10.3389/fnut.2022.1007164)

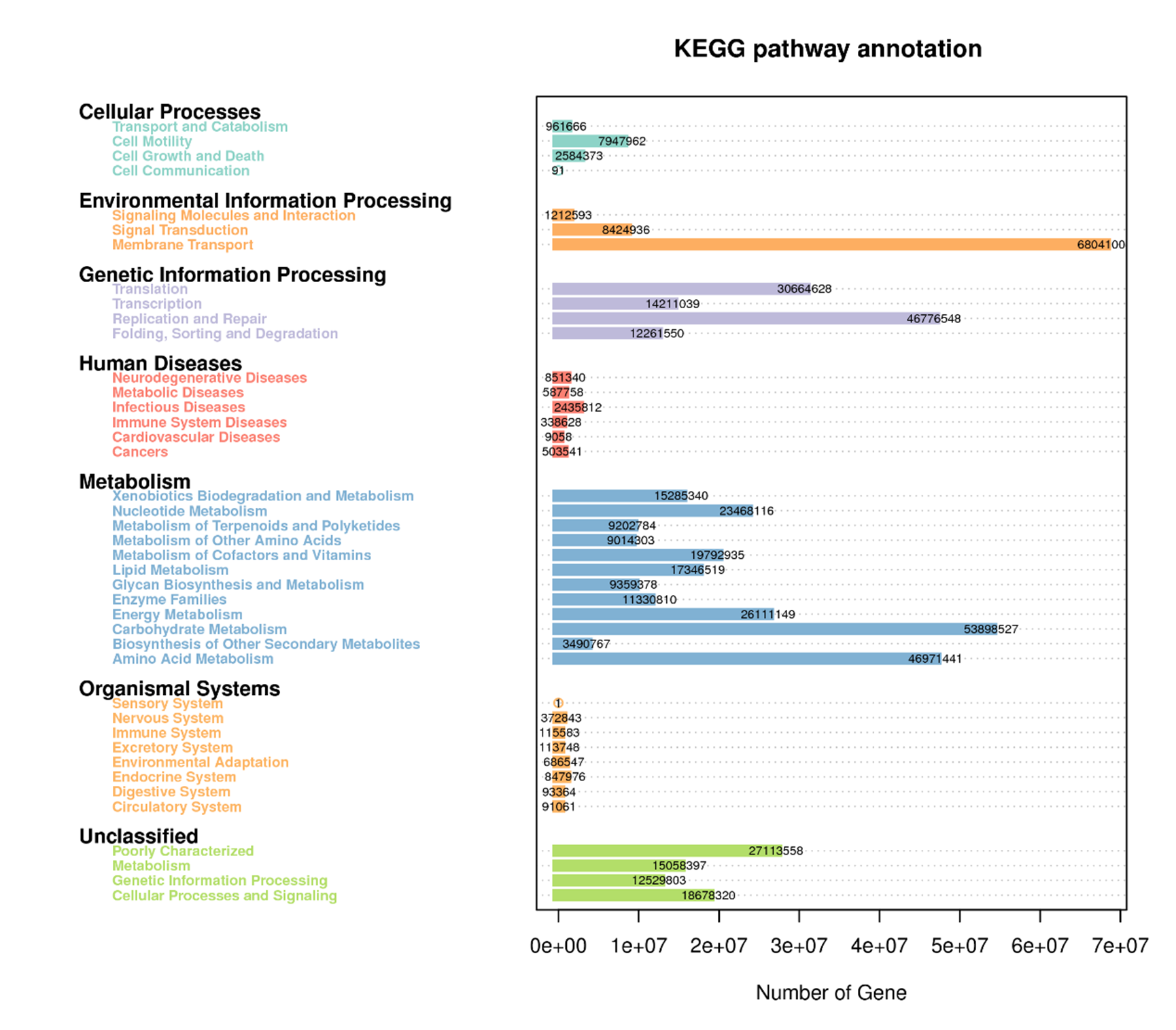

Supplement: Supplementary file 1 [file Image_1.TIF]
